# Simultaneous regulation of miR-451 and miR-191 led to erythroid fate decision of mouse embryonic stem cell

**DOI:** 10.22038/ijbms.2019.27919.6795

**Published:** 2019-04

**Authors:** Gelareh Shokri, Fatemeh Kouhkan, Shahrzad Nojehdehi, Masoud Soleimani, Ali Akbar Pourfathollah, Mahin Nikougoftar Zarif, Mona Tamaddon, Narges Obeidi

**Affiliations:** 1 Stem Cell Technology Research Center, Tehran, Iran; 2 Department of Hematology, School of Medicine, Tarbiat Modares University, Tehran, Iran; 3 Department of Immunology, School of Medicine, Tarbiat Modares University, Tehran, Iran; 4 Blood Transfusion Research Center, High Institute for Education and Research in Transfusion Medicine, Tehran, Iran; 5 Department of Hematology, School of Para Medicine, Bushehr University of Medical Sciences, Bushehr, Iran

**Keywords:** Differentiation, Erythropoiesis, miR-451, miR-191, Mouse embryonic stem cell

## Abstract

**Objective(s):**

Various microRNAs (miRNAs) are expressed during development of mammalian cells, when they aid in modulating gene expression by mediating mRNA transcript cleavage and/or regulation of translation rate. miR-191 and miR-451 have been shown to be critical regulators of hematopoiesis and have important roles in the induction of erythroid fate decision. So, the aim of this study is investigation of the miR-191 and miR-451 roles in the controlling mouse embryonic stem cell (mESC) differentiation toward the erythroid lineage.

**Materials and Methods:**

mESCs were infected with either pCDH-miR-Off-191 viruses in pCDH-miR-Off-191 group or simultaneously with pCDH-miR-Off-191 and pCDH-miR-451 lentiviruses in simultaneous group. Then, the expression profiles of erythroid specific transcription factors and globin genes were analyzed using QRT-PCR on day 14 and 21 of differentiation. Flow cytometry analysis was used to evaluate of TER119 and CD235a erythroid specific surface markers.

**Results:**

Gata-1, Klf-1, Epor and globin chains were found to be expressed in pCDH-miR-Off-191 and in simultaneous groups. The majority of globin chains showed changes in their expression levels with progression of differentiation from day 14 to day 21. Flow cytometry results showed that miR-451 up- regulation and miR-191 down-regulation is associated with the expression of TER119 and CD235a. Of these two groups analyzed, simultaneous group was most significantly potent in stimulation of erythroid fate decision of mESCs.

**Conclusion:**

Together, present data demonstrate that down-regulation of miR-191 alone can enhance the differentiation of mESCs. However, the simultaneous effect of miR-451up-regulation and miR-191 down-regulation is much stronger and can have more practical use in artificial blood production.

## Introduction

Survival without blood is not possible (1). Low blood supplies especially in developing countries, lower number of donors owing to the aging of population and consequently increased demand for blood products, short storage period, and urgent needs for blood supplies during wartime and natural disasters are important reasons that caused the scientists started to think about a proper replacement such as an artificial blood (2, 3). Some kind of blood substitutes developed over time, including red blood cells (RBCs) isolated from donated blood, perfluorochemicals (PFCs), Hb-based RBC substitutes, but the real RBCs are RBCs differentiated from stem cells (4, 5). RBC-derived stem cells used as an ideal product for injection to patients needing blood transfusion as well as patients with rare blood groups or autoantibodies (6). For this reason, stem cells could be derived from various sources such as bone morrow, cord blood, embryonic stem cells, and induced pluripotent stem cells (iPSCs) (7-9). However, there are most important problems at clinical applications including mass production and high production costs for the use of various cytokines and growth factors. It would be expected to overcome these obstacles through genetic manipulations of the molecular mechanism in order to direct the fate decision of the stem cells to erythroid lineage and make an unlimited source with maximum similarity and minimum costs to replace RBCs derived from donated blood. Therefore, the exact study of the molecular processes governing the differentiation of stem cells into blood cells is very much considered. 

In this field, a large body of recent studies has suggested that single-stranded RNA molecules of 21–23 nucleotides, termed as microRNAs (miRNAs), play critical roles in differentiation processes of stem cells into RBC lineage (10, 11). Specific miRNAs are key regulators of each stage of erythropoiesis, including erythroid lineage determination, erythroid progenitor proliferation, terminal erythroid differentiation, and enucleation (12, 13). Jin and coworker demonstrated that expression levels of miR-142-3p, miR-142-5p, miR-146a and miR-451 were dynamically changed during differentiation of hESCs to CD34+ hematopoietic cells, and in subsequent differentiation of the CD34+ cells into the erythroid lineage (14). In the other study the expression profile of 295 miRNAs before and after their induction to erythroid differentiation was analyzed using microarray. Among them, miR-451 was most significantly up-regulated during erythroid maturation (15). Also, Kouhkan *et al*. indicated that expression modulation of miR-451 and miR-150 could be an efficient alternative to stimulatory cytokines for CD133+ differentiation into erythroid lineage (16). Regulated expression of several miRNAs is also important in terminal erythroid differentiation for erythroblast chromatin condensation and enucleation (17, 18). For example, Riok3 and Mxi1, which are required for chromatin condensation and enucleation, are direct miR-191 targets (19). So, ectopic overexpression of miR-191 in mouse fetal liver erythroid progenitors blocked erythroid enucleation but had minor effects on proliferation or erythroid differentiation. 

Since, the major problem of patients with hemoglobinopathies, such as sickle cell anemia and thalassemia is failure in the production of adult globin (HbA), reactivation of the α- and β-globin chains could somewhat improve the lethality of α- and β-thalassemia (20, 21).

So, taking into account the importance of miRNAs in differentiation, the aim of this study is to investigate whether synchronous regulation of miR-451 and miR-191 could emerged as a replacement to the stimulatory cytokines and induced efficient differentiation of mESC into erythroid lineage.

## Materials and Methods

***Cell culture***

Murine ESC (mESC) [E14Tg2A] lines were cultured feeder-free on 0.1% gelatin-coated (Sigma, USA) plates in ESC medium (Dulbecco’s modified Eagle’s medium, 15% heat-inactivated embryonic stem-standard fetal bovine serum [Gibco, USA], 1 mM nonessential amino acids [Gibco, USA], 2 mM L-glutamine [Euroclone, Italy], 55 nM -mercaptoethanol, 1% [vol/vol] penicillin/ streptomycin [Euroclone, Italy], and mouse leukemia inhibitory factor [mLIF; 103 U/ml; Sigma, USA]). 

Human embryonic kidney (HEK) 293 cell lines were obtained from Pasteur Institute of Iran and cultured in high-glucose Dulbecco’s modified Eagle medium (DMEM, Gibco, USA) containing 10% fetal bovine serum (FBS, Gibco, USA) and 1% nonessential amino acids (Invitrogen, USA), penicillin (100 U/ml, Gibco, USA), streptomycin (0.1 mg/ml, Gibco, USA), and L-glutamine (2 mM, Gibco, USA). Cells were cultured under standard condition in 95% humidity and 5% CO_2_ at 37 ^°^C.

***Recombinant lentivirus generation and infection***

For gain of function study, pCDH-miR-451 recombinant lentivector construct was cloned as described previously (22). For loss of function study, shRNA structure of mutant form of miR-191 was cloned into the pCDH-CMV-MCS-EF1-copGFP plasmid to create pCDH-miR-Off-191. Empty vector without any cloned sequence (pCDH-Ctrl) was employed as controls in all experiments. 

For lentivirus production, HEK-293T cells were calcium phosphate co-transfected with pCDH-miR-451/ pCDH-miR-Off-191 or pCDH-Ctrl, pPAX2 plasmid (packaging plasmid) and pMD2 plasmid (containing vsv gene). The supernatants were harvested every 12 hours for 3 days after transfection and concentrated by ultracentrifuge at 47,000×g for 2 hr at 4 ^°^C. 

To induce erythroid differentiation, mESCs were cultured in leukemia inhibitory factor (LIF)-deficient medium and transduced with pCDH-miR-Off-191 and pCDH-miR-451 lentivirus. 

Then, the effect of miR-451 up-regulation and miR-191 down-regulation on erythroid differentiation was measured over a period of 14 days and 21 days. 

***RNA extraction, RT-PCR and quantitative RT–PCR assays***

Total RNAs were extracted from all group of study using TRizol reagent according to the manufacturer’s instructions (Invitrogen, USA). cDNA was synthesized with 200 ng of total RNA using M-MuLV reverse transcriptase (Promega, USA) and random hexamers (for mRNAs) or stem-loop RT specific primers (for miR-451, miR-191 and SNORD234). 

Quantitative real-time PCR was performed using the ABI 157 PRISM 7500 real-time PCR system (Applied Biosystems). Normalization was performed with β2M and SNORD234 for mRNAs and miRNAs genes, respectively. Finally data analyses were performed using 2^−∆∆CT^ method. Primer sequences are given in the supplemental Table 1.

***Flow cytometry***

For erythroid-specific surface markers detection (Ly76: TER119 and glycophorin A receptor: CD235a) mESC samples from all groups were collected at days 14 and 21, washed with PBS and immunostained with purified PE -conjugated antibody against CD235a (1:200), PE-conjugated antibody against TER119 (1:200, BD Pharmingen, USA) and isotype-matched control antibodies (DAKO Denmark) for 60 min at 4 ^°^C in dark. Harvested cells then examined on FACScan flow cytometry PartecPAS III (Partec, Germany).

***Statistical analysis***

All experiments were performed at least three times, presented as means±standard deviation (SD) and analyzed by student’s t test. Values with *P*<0.05 were considered statistically significant.

## Results

***Simultaneous regulation of miR-451 and miR-191 in mESCs promoted erythroid differentiation***

In the previous study, we indicated that pCDH-miR-451 could induce erythtroid fate determination in mESCs (23). According to importance of miR-191 down- regulation in erythroblast enucleation as erythroid specific miRNAs, in the present study, the effects of simultaneous regulation of these two important miRNAs and the effect of miR-191 down-regulation alone were investigated in differentiation induction. 

So, in order to drive mESCs to enter erythroid commitment, cells were transduced with pCDH-miR-451 and/or pCDH-miR-Off-191 expressing copGFP and used to form EBs in suspension culture. Transduction efficiency was checked through monitoring of CopGFP expression by fluorescent microscope. Approximately 80% of the cells in transduced groups with green fluorescent were detected 48 hr after infection. No fluorescent-positive cell was detected in control group that did not receive any treatment, as shown in Figure 2: Expression analysis of erythroid specific transcription factors in transduced mESC on the 14 and 21 day in transduced mESCs. In the absence of cytokines, down-regulation of miR-191 could induce transcription of lineage-specific factors and promote erythroid fate decision. Results presented as fold change compared to the control cells. Data are mean ± SD from three independent experiments. Error bars represent SD. *P<0.05 group (transduced simultaneously with pCDH-miR-451 and pCDH-miR-Off-191) and pCDH-miR-Off-191 group and miR-451 was up-regulated in the simultaneous group as comparing to the control groups (no vector group and pCDH-Ctrl group) after transduction. 

To further confirmation that Riok3 and Mxi1 are direct targets of miR-191 during terminal differentiation of erythroids, we measured Riok3 and Mxi1 transcripts on day 14 and 21 differentiation. Our results indicated that down-regulation of miR-191 increased the expression of Riok3 and Mxi1 (Figure 2A). Additionally, since, miR-451 through direct targeting of 14-3-3 ζ and GATA2 involve in erythroid differentiation, the expression level of these proteins were also examined (Figure 2B). Over-expression of miR-451 specifically reduced 14-3-3ζ and GATA2 mRNA expressions in simultaneous group demonstrating that putative targets transcript levels correlates inversely with miR-451 levels.

Next, to verify the direction of the differentiation path, we investigated the effects of miR-451 and miR-191 modulation on the expression pattern of several erythroid-specific marker genes by quantitative RT-PCR. In the simultaneous group, gene expression analysis showed elevated expression levels of Epor and Klf-1 mRNAs on day 14 and their expressions remained high until the end of experiment. GATA1 mRNA level was up-regulated on day 14 but decreased to 0.36 fold on day 21 (Figure 3A-B). 

In the case of pCDH-miR-Off-191, highest expression level of Epor and Klf-1 were observed on day 21 (with an increase of 6.27 and 38.31 times, respectively, compared to the control group) Gata-1 expression only detected 14 days after differentiation (Figure 3C-D). 

The expression profile of hemoglobin chains were also examined in tansduced groups (Figure 4). mRNA of *ε, γ* and *α* genes were significantly overexpressed in simultaneous group (6.02-fold, 22.31-fold and 17.72-fold respectively), and in pCDH-miR-Off-191 group (3.13-fold, 4.02-fold and 10.55-fold) compared with pCDH-Ctrl control group on day 14. 

At day 21, expression of ε, α and β-globin genes reached its highest level in simultaneous group (by 7.3- fold, 31.6- fold and 45.2-fold, respectively) compared with the untreated control group (*P*<0.05). Knockdown of miR-191 had positive effects on the mRNA expression of ε, α and β-globin genes. There were no significant changes in the expression profile of hemoglobin chain genes in control group (no vector) and pCDH-Ctrl group during the course of differentiation. Collectively, these results confirmed that simultaneous regulation of miR-191 and miR-451 stimulates the expression of genes involved in the erythroid differentiation pathway more potently.

***TER-119 and CD235a were expressed in both pCDH-miR-Off-191 group and simultaneous group***

Expression of TER119 and CD235a consider as key indicators of erythropoiesis. As shown in Table 1, flow cytometry results demonstrated that in the pCDH-miR-Off-191 group, knockdown of miR-191increased the number of the cells expressing TER119 and CD235a (15 ± 3.34% and 10.47± 2.78%, respectively, compared with 3± 1.21% and 2.98 ± 1.52% of the pCDH-Ctrl) on day 14. On day 21, results indicated that the positive cell population for TER119 and CD235a were 21± 2.61% and 18.7± 3.97% in pCDH-miR-Off-191 treated mESCs and 3.90 ± 1.41% and 3.13 ± 1.45% in pCDH-Ctrl group, respectively (Figure 3).

The results of the flow cytometry show that simultaneous regulation of the miR-451 and miR-191 significantly increased the percentage of the cells expressing TER119 and CD235a to 32.25 ± 3.34% and 25.57 ± 2.21% at day 14 and 75.34 ± 3.71% and 65.86 ± 2.64% at day 21 (Figure 4). 

## Discussion

It is clear that much attention has been devoted to the role of miRNAs in erythroid lineage commitment over the past few years (24-26). Previously, it has been thought that the production of erythroid cells are regulated only by cytokines, including erythropoietin, IL-3, IL-6, stem cell factor, glucocorticoids and TPO that activate the JAK/STAT, RAS/ MAPK and PI3K/AKT pathways, all of which finally regulate transcription factors, such as GATA1, TAL1/SCL, STAT5 and KLF1, to eventually modulate the expression of genes required for fate decision and differentiation (27-30). The identification of miRNAs that are required for erythroid cell lineage commitment discovers another layer of ‘decision-making’ process.

In the previous study, we demonstrated that miR-451 plays an important role in promoting erythroid maturation and accelerated the rate of erythroid differentiation of the mESCs, an action mediated in part by repression of GATA2 (23). Here, we indicated that another part of the miR-451’s activity is applied through protects against erythroid oxidant stress by repressing 14-3-3ζ .14-3-3 ζ is a phospho-serine/threonine-binding protein that inhibits nuclear accumulation of transcription factor FoxO3, a positive regulator of erythroid anti-oxidant genes (31, 32).

**Figure 1:**
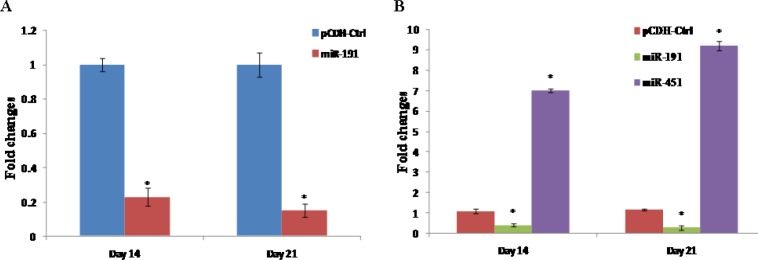
Expression levels of miRNAs in transduced groups on day 14 and 21. A) Expression level of miR-191 in pCDH-miR-Off-191 group. B) Expression level of miR-191 and miR-451 in simultaneous group. Each test was performed triplicate and results were reported as mean±SD of 3 expriments. Error bars indicate SD. *P<0.05

**Figure 2:**
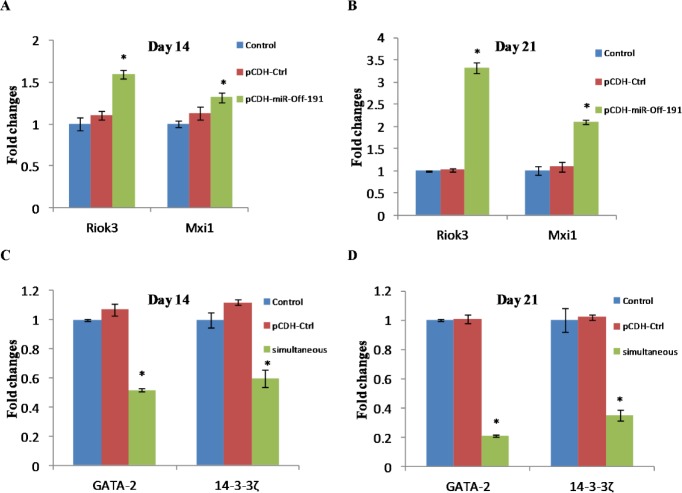
Expression analysis of erythroid specific transcription factors in transduced mESC on the 14 and 21 day in transduced mESCs. In the absence of cytokines, down-regulation of miR-191 could induce transcription of lineage-specific factors and promote erythroid fate decision. Results presented as fold change compared to the control cells. Data are mean±SD from three independent experiments. Error bars represent SD. *P<0.05

**Figure 3:**
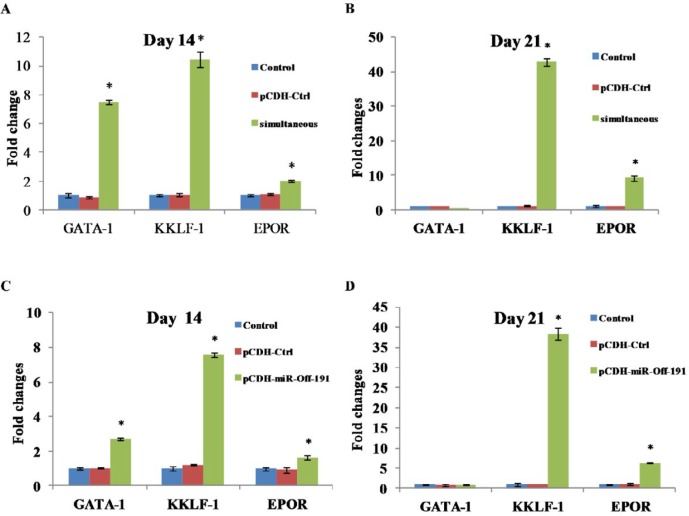
Expression analysis of hemoglobin chains in transduced mESC on the 14 and 21 day. In pCDH-miR-Off-191, down-regulation of miR-191 leads to significant rise of γ and α transcripts on day 14 and 21. Expression of β chain only was observed on day 21. Coordinate modulation of miR-191 and miR-451 had major effect on the transcription of erythroid specific markers and leading to a significant rise of α, γ and β transcripts on day and 14 and 21. Data are mean ± SD from three independent experiments. Error bars represent SD. *P<0.05

**Figure 4:**
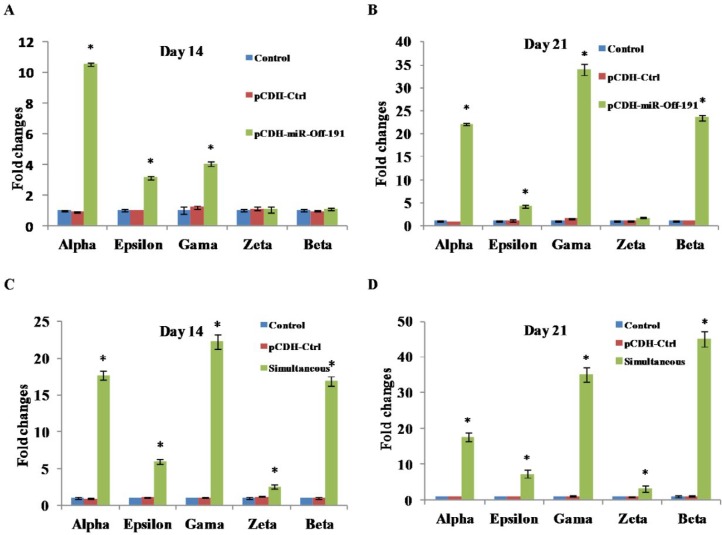
Appearance of TER119 and CD235a in mESCs on day 14 and 21 in pCDH-miR-Off-191 group. Down-regulation of miR-191 had positive correlation with expression of erythroid surface specific marker. A) The percentage of the cells expressing TER119 on day 14 in pCDH-miR-Off-191 group (Upper) and in pCDH-Ctrl group (Lower). B) The percentage of the cells expressing TER119 on day 21 in pCDH-miR-Off-191 group (Upper) and in pCDH-Ctrl group (Lower). C) The percentage of the cells expressing CD235a on day 14 in pCDH-miR-Off-191 group (Upper) and in pCDH-Ctrl group (Lower). D) The percentage of the cells expressing CD235a on day 21 in pCDH-miR-Off-191 group (Upper) and in pCDH-Ctrl group (Lower)

**Figure 5:**
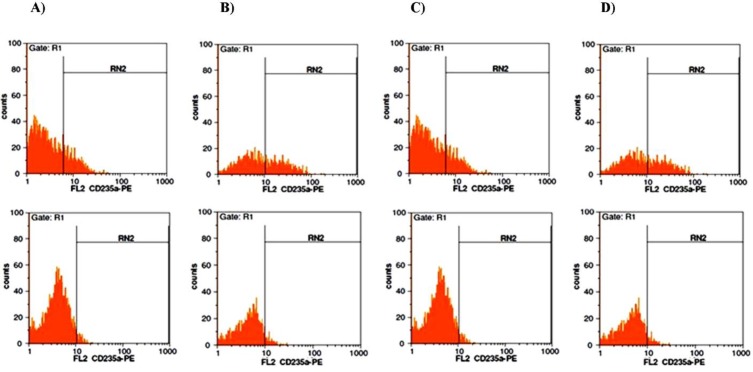
Appearance of TER119 and CD235a in mESCs on day 14 and 21 in pCDH-miR-Off-191 group. Down-regulation of miR-191 had positive correlation with expression of erythroid surface specific marker. A) The percentage of the cells expressing TER119 on day 14 in pCDH-miR-Off-191 group (Upper) and in pCDH-Ctrl group (Lower). B) The percentage of the cells expressing TER119 on day 21 in pCDH-miR-Off-191 group (Upper) and in pCDH-Ctrl group (Lower). C) The percentage of the cells expressing CD235a on day 14 in pCDH-miR-Off-191 group (Upper) and in pCDH-Ctrl group (Lower). D) The percentage of the cells expressing CD235a on day 21 in pCDH-miR-Off-191 group (Upper) and in pCDH-Ctrl group (Lower)

**Figure 6:**
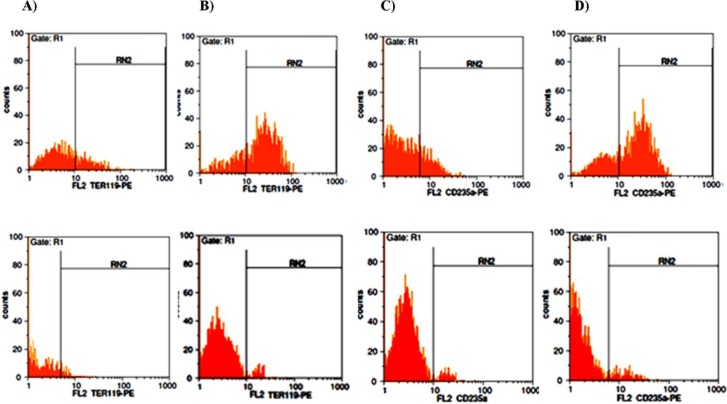
Expression of TER119 and CD235a on the surface of transduced mESCs after 14 and 21 day in simultaneous group. Simultaneous modulation of miR-451 and miR-191 leads to the emergence of TER119 and CD235a. A) The population of the cells positive for TER119 on day 14 in simultaneous group (Upper) and in pCDH-Ctrl group (Lower). B) The population of the cells positive for TER119 on day 21 in simultaneous group group (Upper) and in pCDH-Ctrl group (Lower). C) The population of the cells positive for CD235a on day 14 in simultaneous group (Upper) and in pCDH-Ctrl group (Lower). D) The population of the cells positive for CD235a on day 21 in simultaneous group (Upper) and in pCDH-Ctrl group (Lower)

**Table 1:** The proportion of the cells expressing TER119 and CD235a

Treatment	Day 14	Day 21
The percentage of the cells expressing TER119		
pCDH-miR-Off-191 group		
Coordinate group	32.25±3.34	75.34±3.7
pCDH-Ctrl	3±1.21	3.90±1.41
Control	3.87±0.54	4.07±0.91
The percentage of the cells expressing CD235a		
pCDH-miR-Off-191 group	10.47±2.78	18.7±3.97
Coordinate group	25.57±2.21	65.86±2.64
pCDH-Ctrl	3.84±1.64	6.5±2.43
Control	2.05±0.52	5.32±5.33

On the other hand, terminal erythroid differentiation is a synchronized process that involves four to five cell divisions, expression of many erythroid-important genes, chromatin condensation, and enucleation (33). miR-191 was identified as one of essential down- regulated miRNAs for erythroid chromatin condensation and enucleation by allowing up-regulation of Riok3 and Mxi1(34-36). In the present study, we also confirmed that miR-191 through direct targeting of Riok3 and Mxi1induce erythroid differentiation in mESCs.

So, according to important roles of miR-451 and miR-191, mESCs were transduced with pCDH-miR-Off-191 and/or pCDH-miR-451 and evaluated for emergence of erythroid-specific markers.

Erythroid specific transcription factors evaluation indicated that expression of all three GATA1, FOG-1, and EKLF factors were observed on 14st day of differentiation. FOG-1 and EKLF had a sharp increase on day 21. But, GATA1 expression fell down on day 21 especially in the simultaneous group. In fact, GATA1 is essential for normal erythropoiesis as GATA1-deficient embryonic stem cells are able to contribute to all different tissues in chimeric mice, with the exception of the mature red blood cells. However, at late stages of erythroid differentiation, GATA1 degraded by caspases that leads to a reduction of GATA1 levels and allowing terminal differentiation (37-39).

Further study on expression profile of hemoglobin chains using qRT-PCR indicated that increase accumulation were detected for ε, γ and ζ globin chains transcripts on day 14 and for α and ε globin chains transcripts on day 21 in the pCDH-miR-Off-191 group. β globin was found in the simultaneous group, at a lower level on day 14 and more on day 21 and in the pCDH-miR-Off-191 group, only on day 21. β globin chain expression on the 21^st^ day in the simultaneous group is about twice the pCDH-miR-Off-191 group. These results showed that miR-451 has the dominant effect on the expression of the β gene and producing of adult hemoglobin and further confirmed some previous studies indicating that miR-451 has strong positive association with the late stage of erythropoiesis.

Next, the correlation between miR-451 up-regulation and miR-191 down-regulation with appearance of erythroid specific surface marker as another indexes of erythroid differentiation were investigated. TER-119 is an erythroid-specific cell surface marker that expressed at all stages of differentiation from early proerythroblasts to mature erythrocytes. TER-119 marker was demonstrated to be expressed on day 14 and gradually increased on the 21^st^ day in pCDH-miR-Off-191 group. In this group, CD235a expression was about 10% on day 14, which increased by about 18% on day 21. In the simultaneous group, expression changes were more dramatic: TER-119 marker was expressed about 32% on day 14 and rose to its highest level on the 21st day. CD235a expression increased on day 14 and gradually reached to the peak level on day 21.

## Conclusion

Declined levels of miR-191, to some extent, can enhance differentiation of mESCs alone, where this miRNA exerts its effects through post-transcriptional regulation of multiple mRNAs, including translation attenuation of Riok3 and Mxi1. However, the simultaneous modulation of miR-451 and miR-191 is far more influential in erythroid induction. These findings will aid in our understanding of miRNA function in embryonic stem cells and facilitate our ability to enhance the differentiation of erythroid cell lineages and production of artificial RBCs without the presence of any stimulatory cytokines. 
